# An Empirical Study on Hazardous Chemicals Risk of Urban Residents in China: Analysis of Mediating Effect and Channel Preference of Response Action Decision Model

**DOI:** 10.3390/ijerph182010932

**Published:** 2021-10-18

**Authors:** Ziwei Wang, Yongkui Liu, Tiezhong Liu

**Affiliations:** 1 School of Economics and Management, Beijing Institute of Graphic Communication, Beijing 102600, China; wangziwei@bigc.edu.cn; 2 Chinese Academy of Labour and Social Security, Beijing 100029, China; 3 School of Management and Economics, Beijing Institute of Technology, Beijing 100081, China; liutiezhong@bit.edu.cn

**Keywords:** hazardous chemicals risk, response action decision model, mediating effect, channel preference, structural equation model

## Abstract

Because urban residents do not have a strong understanding of hazardous chemicals, they cannot effectively make response action decisions to ensure safety, protect lives, and reduce property damage. This paper constructs the Response Action Decision Model of hazardous chemicals, and analyzes the mediating effect of Information Processing and Threat Perception, as well as channel preferences of urban residents with different demographic characteristics. A total of 1700 questionnaires were collected in Chongqing, Tianjin, Fujian Zhangzhou, Shandong Zibo and Lanzhou, where there are significant hazardous chemicals factories. The results show that: Firstly, Information Processing and Threat Perception have significant mediating effects on the relationship between Mass Media, Social Media, Face-to-face communication and Response Action Decision in a single channel, which can effectively promote the spread effect of different channels, affecting the ways that urban residents make hazard response action decisions; secondly, Information Processing and Threat Perception do not have a mediating effect on the relationship between the channel combination of “Mass Media ↔ Social Media”, “Mass Media ↔ Face-to-face communication”, “Social Media ↔ Face-to-face communication” and Response Action Decision, and the channel combination can directly link to the Response Action Decision; thirdly, in terms of the extent that it affects urban residents to make response action decisions, Mass Media is greater than Social Media and greater than Face-to-face communication; fourthly, two demographic characteristics of gender and experience have a stronger moderating effect for the Mass Media channel, while other demographic characteristics have greater influences on the Response Action Decision Model; finally, the Response Action Decision Model can be better applied to those analyses and research which address threat perception of hazardous chemicals and response action decisions of urban residents in China.

## 1. Introduction

### 1.1. Research Background

The safety management of chemical hazards is a major global concern. Since the 1950s, there have been more than 60 major environmental pollution incidents occurring around the globe, causing sickness in 400,000 to 500,000 people, and the death of 100,000 [1]. Since the 1980s, China has made remarkable achievements in many fields, such as the economy, culture, science and technology, industry, etc. With further development of the chemicals industry, the use of hazardous chemicals has become more frequent [2]. As a result, a series of major accidents have occurred in recent years, such as the PX project explosion in Gu Lei Peninsula [3], the “11.22” oil pipeline leakage and explosion accident in Qingdao City [4], the “8.12” hazardous chemical warehouse fire and explosion accident in Tianjin Port [5], etc. These incidents all reflect a weak perception of the threat of hazardous chemicals in urban residents, and that urban residents do not respond appropriately, resulting in more casualties and greater property damage. The main reasons are as follows: On the one hand, because the media (including official media, social media, mass media, etc.) does not fully publicize hazardous chemicals, urban residents do not have an avenue to obtain more hazardous chemical information; on the other hand, urban residents do not pay enough attention to hazardous chemicals, which are unfamiliar to them in comparison to e.g., fire, earthquakes and other hazards, deeming that the danger is far removed from them. This prohibits the urban residents from taking the initiative to accumulate relevant knowledge of hazardous chemicals, thus lowering their threat perception and knowledge of the appropriate actions to take in response to a hazardous chemical event.

### 1.2. Literature Analysis

For the first reason, research by foreign scholars has focused on the analysis of different types of information channels. For example, Lindell and Perry (1987, 1992) [6,7], found that warning information can be transmitted through face-to-face contact, telephone, alarm, mobile speaker, radio and other channels; Hance, Chess, Sandman (1988) [8] and Mileti, Sorensen, O’Brien (1992) [9] believed that the main risk communication channel for the community is electronic media; Perry and Nelson (1991) [10] argue that, subject to personal preferences, the community needs to carry out cross-channel risk communication; Julian Conrads and Tommaso Reggiani [11] adopted an experimental economics framework, which investigated the effect of different communication channels on promise-making and promise-keeping in an organizational context, i.e., face-to-face, phone call, chat room, and two different sorts of computer-mediated communication. In. research by domestic scholars, Xie, Li, Yu (2008) [12] proposed that TV can arouse higher risk perception than that of web pages, after comparing the influences of two communication channels on risk perception, while Xue, Wang, Yu (2015) [13] and Ren (2016) [14] mainly focused on the influences of micro-blogs, social media and other emerging medium on the risk perception of the PX project. It can be concluded then, that as the effect of different channels on the transmission of hazard information is different, a comprehensive evaluation concerning the characteristics of communication channels, the types of disasters, the location of disasters, the clues of disasters, and the cultural characteristics of the people at risk should be made to inform the selection of the appropriate communication channels to disseminate hazard information.

For the second reason, even when urban residents have obtained information on the hazards posed by hazardous chemicals from a variety of channels, they may still lack the knowledge on how to respond after perceiving a threat from hazardous chemicals, due to the lack of current academic research in this area. Some scholars have studied hazardous chemicals, and tend to focus on areas such as hazardous chemical management (Skrehot, P.A. et al. [15], Sun and Olivia [16], Scruggs, C.E. [17], Su Dayong, et al. [18], Liu Hong et al. [19]), the transport of hazardous chemicals (Poechlauer P. et al. [20], Kumar, D. et al. [21], Goh, Cb. et al. [22], Meng Song et al. [23], Zhang Jianghua and Zhao Lajun [24], Wang Jun and Chu Yanling [25]), different hazardous chemical treatments (Kumar, D. et al. [26], Patricio Silva, A.L. et al. [27], Singh, R. et al. [28], Xu Ji [29], Qian Yong [30]), and related research methods involving hazardous chemical processes (Jacobs, M.M. et al. [31], Yuan, L. et al. [32], Bao, C.L. et al. [33], Di Jianhua and Zhen Liang [34], Zhang Wenhai et al. [35], Zhang Chao et al. [36], Li Shuanglin [37], Jing Ke and Tang Liang [38]). In order to fill the research gap, this study uses a structural equation model to construct the multi-stage model of “Channel Preference→Information Processing→Threat perception of hazardous chemicals→Response Action Decision” based on the theory of the Protection Action Decision Model (PADM) [39], and further analyzes how external factors such as channel preferences affect the threat perception of urban residents, and thus affect their response to the hazard. Meanwhile, the PADM model was modified to analyze the direct relationship between the external factors and the response, and the mediating effect of Information processing and Threat perception of hazardous chemicals.

### 1.3. Theory and Hypothesis

The Protective Action Decision Model (PADM) is a multi-stage model that is based on research into people’s responses to environmental hazards and disasters. The PADM integrates: first, the processing of information derived from social and environmental cues with messages that social sources transmit through communication channels to those at risk (Lindell and Perry, 2012 [39]), emphasizing that people exposed to a potential risk receive risk information from outside, and that the resulting risk perception is derived from the combination of that information and their preexisting beliefs based on their past knowledge (Wei et al., 2016 [40]); second, the PADM proposes that stakeholder (especially information sources) characteristics directly affect people’s perceptions of hazard characteristics which, in turn, can affect risk perception (i.e., expected personal impacts), and, ultimately, their recognition of evacuation (Huang et al., 2012 [41]); third, the PADM describes a set of mediated relationships that could explain the variation in effect sizes that were found in the statistic meta-analysis (Huang et al., 2017 [42]). PADM is a theoretical model that needs to be applied to different types of disasters in order to be improved. In recent years, the PADM has been used in evacuation during the Three Mile Island crisis, citizen’s perceptions of flood hazard adjustments, and hurricane evacuation, as well as consumer behavior and psychology in Volkswagen recall cases. When applied, some scholars have taken the PADM directly as the theoretical basis to support the research objective, while others have improved the PADM based on other theories, constructing a new decision or evaluation model. However, the PADM does not specify whether channel preferences or other external information can directly affect the protective decisions that people make, or explain the mediating effect of pre-decision processing and threat perception in this multi-stage model. Given the above theoretical analysis, this study attempts to apply PADM to the field of hazardous chemicals. By presenting 12 research hypotheses, after collecting and analyzing questionnaire data, it is verified whether the following assumptions are correct in the description of the relationship between Channel Preference, Threat Perception, and Response Action Decision.

**Hypothesis** **1** **(H1).**  *The communication channel can directly establish a relationship with the Response Action Decision, that is, it does not need to be processed by the Information Processing and the Threat Perception of hazardous chemicals so that the urban residents can make response action decision*.

**Hypothesis** **2** **(H2).**  *The communication channel can establish a direct relationship with the Threat Perception of hazardous chemicals, without going through the stage of Information Processing*.

**Hypothesis** **3** **(H3).**  *Information processing can directly establish a relationship with the Response Action Decision without going through the phase of Threat Perception*.

**Hypothesis** **4** **(H4).**  *Information processing and Threat perception can both exert a mediating effect on different channel preferences*.

**Hypothesis** **5** **(H5).**  *While different sex groups have the same preference for the same channel→ Information Processing→Threat perception of hazardous chemicals→Response Action Decision, the two groups have different preferences for different channels→Information processing→Threat perception of hazardous chemicals→Response Action Decision*.

**Hypothesis** **6** **(H6).**  *While different marital status groups have the same preference for the same channel→Information Processing→Threat perception of hazardous chemicals→Response Action Decision, these groups have different preferences for different channels→Information processing→Threat perception of hazardous chemicals→Response Action Decision*.

**Hypothesis** **7** **(H7).**  *While different age groups have the same preference for the same channel→ Information Processing→Threat perception of hazardous chemicals→Response Action Decision, these groups have different preferences for different channels→Information processing→Threat perception of hazardous chemicals→Response Action Decision*.

**Hypothesis** **8** **(H8).**  *While different education groups have the same preference for the same channel→ Information Processing→Threat perception of hazardous chemicals→Response Action Decision, these groups have different preferences for different channels→Information processing→Threat perception of hazardous chemicals→Response Action Decision*.

**Hypothesis** **9** **(H9).**  *While different income groups have the same preference for the same channel→ Information Processing→Threat perception of hazardous chemicals→Response Action Decision, different groups have different preferences for different channels→Information processing→Threat perception of hazardous chemicals→Response Action Decision*.

**Hypothesis** **10** **(H10).**  *While different disaster training experience groups have the same preference for the same channel→Information Processing→Threat perception of hazardous chemicals→Response Action Decision, these groups have different preferences for different channels→Information processing→Threat perception of hazardous chemicals→Response Action Decision*.

**Hypothesis** **11** **(H11).**  *While different geographical location groups have the same preference for the same channel→ Information Processing→Threat perception of hazardous chemicals→Response Action Decision, these groups have different preferences for different channels→Information processing→Threat perception of hazardous chemicals→Response Action Decision*.

**Hypothesis** **12** **(H12).**  *RADM can be applied to research and analysis of threat perception and response action decisions for hazardous chemicals for urban residents in China*.

## 2. Materials and Methods

### 2.1. Theoretical Model

First, three typical mass media communication channels (Lindell and Perry, 1987, 1992, Hance et al., 1988, Hwang, Sanderson, Lindell, 2001, Liu, Zeng, 2011, Cui, 2009) [6,7,8,43,44,45,46], social media (Yuan, 2014, Xie, 2010) [47,48] and face-to-face communication (Zhu, 2009) [49] were chosen after determining channel preference as an independent variable; second, Information Processing and Threat Perception of hazardous chemicals were determined to be mediator variables; third, the Response Action Decision was determined as the dependent variable. Hence the “Channel preference→Information processing→Threat perception of hazardous chemicals→Response Action Decision” multi-stage model was designed as follows:

**Sub-model 1**: Mass media→Information processing→Threat perception of hazardous chemicals→Response Action Decision

**Sub-model 2**: Social media→Information processing→Threat perception of hazardous chemicals→Response Action Decision

**Sub-model 3**: Face-to-face communication→Information processing→Threat perception of hazardous chemicals→Response Action Decision

**Sub-model 4**: “Mass media ↔ Social media” →Information processing→Threat perception of hazardous chemicals→Response Action Decision

**Sub-model 5**: “Mass media ↔ Face-to-face communication”→Information processing→Threat perception of hazardous chemicals→Response Action Decision

**Sub-model 6**: “Social media ↔ Face-to-face communication”→Information processing→Threat perception of hazardous chemicals→Response Action Decision

**Figure 1:**
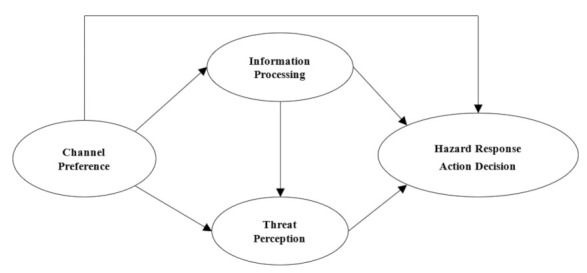
Schematic diagram of multi-stage decision model.

The six models consider the relationship between Information Processing and Response Action Decision, Channel preference and Threat perception, so as to observe the mediating effect of Information Processing and Threat perception on how the urban residents are biased towards different communication channels from which to obtain hazard information and enhance threat perception of hazardous chemicals, which affects their response action decisions. The schematic diagram of the multi-stage decision model is shown in Figure 1.

### 2.2. Variable Measure

A measurement index was designed according to the independent variable, dependent variable and mediator variable. Firstly, for the independent variable, “Channel preference”, the four different measurement indexes (Lindell and Perry, 2004) [50] of preference, permeability, accuracy, and stability were given. In this case, permeability refers to the scope within which information can be disseminated, and the extent to which daily life is disturbed by hazard information; accuracy refers to the authority of information transmitted; stability means whether the spread of information can distort the situation, namely the degree of being understood. Secondly, for the mediator variable of “Information processing”, indexes of exposure, concern, and comprehension were used to conduct the measurement. Thirdly, for the mediator variable of “Threat Perception of hazardous chemicals”, the three indexes of possibility, seriousness, and fear & unknown factors were given, in which possibility refers to the likelihood of danger considered by people to occur around them; seriousness refers to the degree of impact brought to people after any danger occurs; fear & unknown factors refer to people not knowing what will happen, or refer to their degree of fear of some kinds of risk (people know some risk will happen). Fourthly, for the dependent variable of “Response Action Decision”, five indicators were given, which are risk assessment, hazard response action search, hazard response action assessment, hazard response action implementation and information search activity. See variable measurement index and explanation in Table 1.

#### 2.2.1. Channel Preference

Different channels have different characteristics. In China, hazard information from “mass media” needs to be edited and approved by the relevant supervisors at all levels before it can be presented in front of the readers. Because the threshold of “social media” is lower, the transmission speed is faster. The characteristic of “face-to-face communication” shows that the information needs to be issued by higher authorities to the communities which would then organize residents to carry out the theoretical training of relevant hazard knowledge. It can be concluded the selection of different information-spreading channels will engender corresponding effects on the characteristics of the hazard information. Moreover, various levels of residents often choose their preferred channels to obtain hazard information.

#### 2.2.2. Information Processing

The hazard information transmitted from different channels does not directly affect the risk perception of urban residents unless they can receive and pay attention to the hazardous information and understand its content. While the relationship between the communication channel and the threat perception cannot be directly established, the mediating variable of “Information processing”, measured by exposure, attention, and understanding, can effectively enhance the effect of threat perception, and the size of its mediating effect can be tested.

#### 2.2.3. Threat Perception

When presented with hazard information, different population groups show a significant difference in hazard response actions. While professionals often carefully analyze the reliability of information sources and the authenticity of information content, ordinary residents rely more on their own experience, or peer exchange, to confirm whether the information is credible, the possibility of occurrence, and the seriousness of the consequences of disasters. At the same time, some studies have found that fear and unknown factors also affect people’s perception of risk. Thus, differences in the perceived levels of hazard information can affect subsequent hazard response actions.

#### 2.2.4. Mediator Variable

The mediator variable can be explained as the internal mechanism of the relationship between the independent variable and the dependent variable, i.e., how is the relationship as an intermediate conversion role in the process of the “independent variable→mediator variable→dependent variable” affected.

#### 2.2.5. Response Action Decision

This is a crucial stage after Threat Perception, and is informed by the degree of threat perception through further research and confirmation of hazard information, which helps people decide when, where and how to make the appropriate response action decision. This phase is particularly important in the whole response process, and if the threat perception cannot be efficiently transformed into a hazard response action, the efficiency and significance of all previous stages will be significantly reduced.

### 2.3. Sample Selection

#### 2.3.1. Sample Area Selection

According to data from the National Bureau of Statistics on the level of urbanization development (urban population/resident population) [51], the 31 provinces of China can be divided into four files. The ratio of Tianjin is 0.8 or more, the ratio of Jiangsu, Fujian, Chongqing is 0.6~0.8, the ratio of Shandong is 0.5~0.6, and the ratio of Gansu is 0.5 or less. At the same time, according to the quantity distribution of 60 hazardous chemical production safety key cities (districts, counties) set up by the State Council Security Committee [52], data from the State Administration of Safety Administration website [53], and data from the hazardous chemicals incident information network [54], the central and eastern regions are densely populated areas, especially in the Yangtze River Delta, Pearl River Delta, Beijing-Tianjin-Hebei and other large urban groups. These are also places which have the most concentrated distribution of hazardous chemicals with the highest potential risk level. The vast majority of the 33,625 hazardous chemicals companies are located on the eastern side of the Aihui-Tengchong Line, which is a densely populated region in eastern and central China, and is positively correlated with urban distribution and population distribution [55].

Based on the above analysis, six cities in Lanzhou, Fujian, Zhangzhou, Zibo, Changzhou, Chongqing and Tianjin were selected as the target areas. In terms of geographical location, the six selected cities were delegated separately as the representative fortress cities on behalf of the northwest, southwest, southeast, coastal, Yangtze River Delta, Beijing-Tianjin-Hebei areas.

Statistics concerning the keyword “hazardous chemicals” in a Baidu map shows that these six places have more hazardous chemicals enterprises compared with other cities in the same province or other municipalities, and the probability of the distribution of hazardous chemicals enterprises around the residential area is large. It is therefore very scientific, rational and targeted to study the related issues of risk perception of hazardous chemicals in this stratified population.

#### 2.3.2. Sample Survey Method

For the sample survey method, the academic research is more abundant. Wu Jing, Hong Guang [56] proposed a sample survey based on the probability theory, which involves surveying part of a unit and using it to calculate the overall statistics. This method is a central method in the statistical survey method system, with the theoretical basis of the law of large numbers and the central limit theorem. Wei Zhenjun [57] introduced the sample survey method in detail, with the United States draw conscription as an example, describing methods from simple random sampling to representative sampling, up to stratified sampling. Wang Xiaoxia [58], after comparing the effects of equidistant sampling with simple random sampling, isometric sampling with stratified sampling, cluster sampling with simple random sampling, stratified sampling with simple random sampling, and analyzing the effects under different conditions, put forward the measures and evaluation principles applicable to reduce their individual errors in practical work. Ma Defeng [59] suggested that the factors influencing the sampling survey include the representation, operability, quantity, and normality of sample extraction. Shen Hongmei [60] put forward some suggestions to improve the statistical survey method in view of the problems in the practical sample survey. Zhang Mimi et al. [61], after summarizing the progress of the sample survey method in China, added three new methods: multi-stage sampling, double sampling, and PSS sampling on the basis of the original. Yang Lulu [62] gave an overview of the sample survey method, analyzed the status of the sample survey method in statistical work with the opportunities and challenges of the large data age, discussed and studied the precautions needed to carry out sampling surveys effectively, and provided some ideas for research on the status of the sample survey in the present day.

Based on the above theoretical analysis, combined with this sample survey, cities with more hazardous chemicals enterprises should be selected. At the same time, in terms of age and education level constraints, targeted objects should be selected according to different age and education level, using the stratified sampling method. According to Wang Xiaoxia’s analysis, in terms of the stratified sampling method, the greater the deviation of the average of the layers around the overall average, the smaller the stratified sampling error is. It is necessary to narrow the difference in each layer as much as possible, so as to widen the difference between the layers and increase the deviation of the average of the layers on the overall average to obtain a better-stratified sampling estimation effect.

If the overall *N* is divided into *n* layers, the stratified sampling variance is $V\left(\bar{X_{st}}\right)=\frac{\sigma_{wst}{}^{2}}{n}\;\left(1-\frac{n}{N}\right),$ and $\sigma_{wst}{}^{2}$ is the average of the variance in each layer.

Cheng Dachao [63] applied the characteristics of stratified sampling to calculate the re-offense rate of released prisoners. Qiu Saibing and Tang Bo [64] provided a scientific and more accurate random sampling method and its statistical calculation formula for multiple selection sensitivity problems. Su Jie and Guo Jikun [65] constructed a military sampling survey model based on stratified sampling and set pair analysis, and applied it to the practice of sample surveying in a rear oil depot. The practical application of the above stratified sampling method provides a more solid theoretical and practical foundation for the selection of sampling samples in the questionnaire. The research group also analyzes the sample area and sample size according to the above theory.

#### 2.3.3. Sample Size

Aguila and Gonzalez-Ramirez [66] suggested that the definition of sample size is necessary when designing any research plan. The study shows that it is most efficient to sample according to time and cost. Therefore, the determination of the sample size is essential. For finite population, select the calculation formula for the sample size:

(1) $$n=\frac{t_{a}^{2}*p*q*N}{\left(N-1\right)\;*\;e^{2}+t_{a}^{2}*p*q}$$

where *n* = the required sample size; *N* = the total number of selected sample regions; *p* = the desired percentage of the response variable; *q* = 1 − *p*; *e* = acceptable error boundary (typically between 5% and 10%); *t_a_* = normal curve value associated with self-confidence level, e.g., a value of 2.57 for the 99% confidence level, a value of 1.96 for the 95% confidence level, and a value of 1.64 for the 90% confidence level.

For infinite overall population, the selected sample size is calculated as:

(2) $$n=\frac{t_{a}^{2}*p*q}{e^{2}}$$

According to the number of residents in six cities and formula (1), assuming that the confidence level is 95%, *p* = 80%, *e* = 0.05, the number of resident population and the required sample size are shown in Table 2.

The value of *n* is taken as an integer, the sample size of six cities requires 246 people, that is, each city needs to issue 246 questionnaires. The online survey platform “SO JUMP” was entrusted to carry out the sampling survey. The platform sends questionnaires mainly through social media, mass mailing, and other channels. Throughout the process, the user IP address was considered, and a sending spacing was set according to the questionnaire quantity demand, so as to make sure that the questionnaire contains a certain randomness characteristic. Considering that the questionnaire data may contain abnormal values and forgery, and the sample size had some leeway, with six cities issued a total of 1800 questionnaires, among which 100 questionnaires were discarded due to incomplete data. Among these 1700 questionnaires, there were 94% valid values, which meant that the sample size was met. The overall demographic characteristics of the participants are shown in Table 3, including gender, marriage status, age, education, income, and hazards experience.

### 2.4. Data Processing

#### 2.4.1. Reliability Analysis

The Cronbach model was employed to analyze reliability. When entering the data into SPSS 13.0 software, the Cronbach’s Alpha value was 0.922, while the standardized Cronbach’s Alpha value was 0.913. The two coefficient values are greater than 90%, indicating that the data has a high intrinsic consistency and high reliability.

#### 2.4.2. Nonparametric Test

Nonparametric testing was carried out using a chi-square test. The relevant test statistics show that the *p* values for all questions are less than 0.05, except for the categorization of gender, and the question “what is your attitude toward living around a new dangerous chemicals project?”, which had *p* values of 0.244 and 0.255, respectively. The data in this study obey normal distribution.

#### 2.4.3. Exploratory Factor Analysis

Exploratory factor analysis was calculated by SPSS, and the value of *KMO* was 0.912, indicating that it was suitable for factor analysis. The original hypothesis of the Bartlett sphericity test is that the correlation coefficient matrix is a unit matrix; the value of *Sig* is 0.000, which is less than the 0.05 significance level, thus rejecting the original hypothesis, indicating that there is a correlation between variables and is suitable for factor analysis.

The results show that the variance of the variables in the factor analysis is relatively high, indicating that most of the information in the variables can be extracted by the factor, proving that the results of the factor analysis are valid. The eigenvalues of the first 27 factors are greater than 1, and the sum of the eigenvalues of the first 27 factors accounts for 59.7% of the total eigenvalues.

#### 2.4.4. Homology Deviation Test

The sample test was fulfilled with software SPSS 12.0 (IBM, Armonk, NY, USA) to test whether the overall population of the six sample cities are from the same city, and if the six cities have the same distribution. Using the Kruskal–Wallis test and the Jonckheere–Terpstra test, the values of progressive significance in the data are less than 0.05, indicating that there are significant differences in the data from the six cities. In general, the Kruskal–Wallis test is used to test whether multiple independent samples are from the same population, and the median test and the Jonckheere–Terpstra test are used to test whether the different overall populations have the same distribution. Based on the above analysis, the variables involved in the multi-stage model are summarized and analyzed.

## 3. Results

### 3.1. Impact of Characteristics of the Small World on Information Dissemination

Because the core variables involved in the study are unobservable variables, it is necessary to measure by observable index. The structural equation model (SEM) was selected to research according to the characteristics of variables. SEM is a statistical technique which combines “factor analysis” and “regression analysis of linear models” in traditional multivariate statistical analysis, which can then conduct identification, estimation, and verification, etc., on various causal models, and is applicable for the analysis of the complex relationship between variables [67,68]. The technique first uses the survey data to simulate the deduction, and obtain the fitting index values of various models, then it applies the Amos21.0 to carry out confirmatory factor analysis (CFA) on the multi-stage model, and evaluate its construct reliability and validity. This study uses the statistics of composite reliability and AVE (Average Variance Extracted) to evaluate the reliability of constructs. The results are shown in Table 4.

Table 4 shows that *Factor Loadings* are almost all greater than 0.6, and that no negative *SE*, *Composite Reliability* is greater than 0.8; *AVE* is greater than 0.5, showing that the fitting index of the model is better at carrying out the analysis of mediating effects.

### 3.2. Analysis of Mediating Effects

#### 3.2.1. Mediating Effect Analysis of Sub-Models 1–3

**Figure 2:**
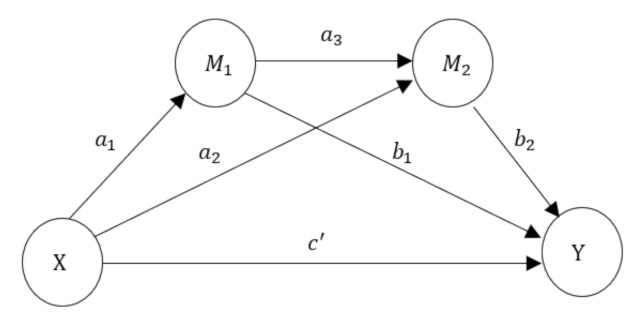
Schematic diagram of multiple mediation models.

Sub-model 1 includes *MM* as independent variables, *IP* and *TP* as the mediator variables, the *RAD* as the dependent variable, with the *IP* and *TP* as the mediator variables between *MM* and *RAD*. Sub-model 1 is a multiple mediation model [69,70], as shown in Figure 2. With the *MM* as the information source, it is necessary to discuss the mediating effect of *IP* and *TP*. Among them, *X* represents the *MM, Y* represents the *RAD*, $M_{1}$ represents the *IP*, the $M_{2}$ represents the *TP*. When the independent variable *X* is replaced respectively by *SM* or *FTF*, it would constitute sub-model 2 and sub-model 3.

The analysis was carried out from three perspectives: firstly, the mediating effect of the specific path, such as $a_{1}b_{1},\;a_{2}b_{2}$ and $a_{1}a_{3}b_{2}$; secondly, the total mediating effect, i.e., $a_{1}b_{1}+a_{2}b_{2}+a_{1}a_{3}b_{2}$; thirdly, the comparison of the mediating effect, such as $a_{1}b_{1}/a_{1}b_{1}+a_{2}b_{2}+a_{1}a_{3}b_{2}$, $a_{2}b_{2}/a_{1}b_{1}+a_{2}b_{2}+a_{1}a_{3}b_{2}$ and $a_{1}a_{3}b_{2}/a_{1}b_{1}+a_{2}b_{2}+a_{1}a_{3}b_{2}$ (Wen et al., 2005) [71].

The tests are based on the data after centralization processing; since all the fitting indexes meet the requirements, the mediating effect analysis can be carried out; Amos21.0 is used to analyze the sub-models 1–3. The statistical results are shown in Table 5.

From the statistical results in Table 4, we can see that the relationship between *IP* and *RAD* is not significant in the three models, and the regression coefficients are negative. At the same time, the regression coefficients between the independent variables and *TP* are both negative, and the *FTF* is not significant. After directly establishing the relationship between the independent variables and *TP*, the results show that the relationship between *MM*, *SM* and *TP* was not significant, and the *FTF* was significant. The changes in the relationship between *FTF* and TP show that in the phrase of *FTF*→*IP*→*TP*, *IP* has a full mediating effect, that is, the transferring effect of *FTF*→*IP*→*TP* is same as the effect of *FTF* which directly establishes the relationship with *TP*. In order to determine the mediating effect of *IP* between the three channels and *RAD*, and the mediating effect of *TP* on *FTF* and *RAD*, we need to use the formula: $Z=\frac{\hat{a}\hat{b}}{\sqrt{{\hat{a}}^{2}s_{b}{}^{2}+{\hat{b}}^{2}s_{a}{}^{2}}}$ to carry out the Sobel test, where $\hat{a}$ is the coefficient of the no significant independent variable, $\hat{b}$ is the coefficient of the corresponding mediator variable; *a*, *b* is the standard error. According to the relevant test standard [72], if $\left|Z\right|$ > 0.97, then it is considered significant. Calculating that *Z_MM-IP_* = 0.27, *Z_SM-IP_* = −1.33, *Z_FTF-IP_* = −1.68, *Z_FTF-TP_* = −0.16, the relationship between *SM*, *FTF* and *IP* is significant, and the relationship between *MM* and *IP*, *FTF* and *TP* is not significant. According to the above determination results, the mediating effect analysis was performed on the sub-models 1–3, and the results are shown in Table 6.

Sub-model 1: The direct relationship between *MM* and *TP* is not significant, however, both coefficient *a* and *b* are significant after taking *IP* as a mediator variable, so in this model, *IP* is a complete mediator variable. Currently, in the literature on the “complete mediating effect”, Wen, Ye (2014) [73] believed that when the direct effect is not significant, it indicates that there is only a mediating effect. When the direct effect is significant, and if the regression coefficients of the two parts have the same number, it is partial mediating effect. On the contrary, it belongs to masking effect. That is, in sub-model 1, hazard information spread by *MM* through *IP*, can better improve the degree of threat perception of urban residents for hazardous chemicals. In the multiple mediation models, as it is not meaningful to consider the full mediating effect of a single mediator variable, it is necessary to analyze whether the mediator variables are significant in the model [74]. In sub-model 1, the independent mediation effect of *IP* is not significant; the independent mediation effect of *TP* is significant, showing that *TP* has a negative effect on urban residents making *RAD* caused by the transmission of hazard information with *MM*; the overall mediating effect of *IP* and *TP* is significant, which can effectively promote the effect of *MM* spreading information on *RAD*; the measure of most significant impact on *IP* is exposure, i.e., the probability of obtaining hazard information on hazardous chemicals (*β* = 0.69, *t* = 19.55), and the measure of the greatest impact on *TP* is the severity (*β* = 0.55, *t* = 23.75).

Sub-model 2: The independent mediation effect of *IP* is significant, which proves that: *IP* has a negative effect on urban residents making *RAD* caused by the transmission of hazardous information with *SM*; the independent mediation effect of *TP* is significant, indicating that *TP* has a negative effect on the relationship between *MM* and *RAD*; the overall mediation effect of *IP* and *TP* is significant, which can effectively promote the effect of *SM* spread information on the *RAD*. The measure of most significant impact on *IP* is exposure, i.e., the probability of obtaining hazard information of hazardous chemicals (*β* = 0.70, *t* = 19.07), and the measure of the most significant impact on *TP* is the severity (*β* = 0.55, *t* = 23.75).

Sub-model 3: The independent mediation effect of *IP* is significant, which proves that *IP* has a negative effect on urban residents making *RAD* caused by the transmission of hazards information with *FTF*; the independent mediation effect of *TP* is not significant; the overall mediation effect of *IP* and *TP* is significant, which can effectively promote the effect of *FTF* spread information on *RAD*. The measure of the greatest impact on information processing is exposure (*β* = 0.70, *t* = 19.72), and the measure of the greatest impact on *TP* is the severity (*β* = 0.55, *t* = 23.75).

Therefore, the hazard information spread by *MM*, *SM* and *FTF* must pass the *IP* and enhance the degree of *TP* of the urban residents in order to make the corresponding *RAD*. The relationship between the independent variables and the dependent variables is significant, which shows a positive correlation in the 3 sub-models. Furthermore, according to the method of analyzing the mediating effect in multiple mediation models [70,73,75], the overall mediating effect is the ratio of the total mediating value to the regression coefficient between the independent variable and the dependent variable. Table 4 shows that the overall mediation effect of the sub-model 3> sub-model 1> sub-model 2, that is, *MM*→*IP*→*TP*→*RAD* has the best effect.

#### 3.2.2. Mediating Effect Analysis of Sub-Models 4–6

Integrated sub-models 1–3 and the independent variables of *MM*, *SM*, *FTF* associate with each other, forming the channel combinations of “*MM* ↔ *SM*”, “*MM* ↔ *FTF*”, “*SM* ↔ *FTF*”, which constitute the sub-models 4–6. Each sub-model is a multiple mediation model whose independent variable has an interaction effect, carrying out analysis according to the method and the step of mediating effect analysis with the latent variable with interaction effect [74,76,77,78,79].

##### Step 1: Interaction Analysis

According to the requirements of Algina and Molder’s [80] modified Joreskog–Yang model [81,82], the measurement indexes of *MM*, *SM* and *FTF* are respectively centrally processed, and MM1*SM1, MM2*SM2, MM3*SM3, MM4*SM4 represent the four measure indicators of MM*SM. The measurement indexes of MM*FTF and SM*FTF were designed with the same way.

MM*SM→*RAD*, significant (*β* = −0.17, *t* = 16.46, *p* < 0.001); MM*FTF→*RAD*, significant (*β* = −0.13, *t* = 16.40, *p* < 0.001); SM*FTF→*RAD*, significant (*β* = −0.09, *t* = 11.29, *p* < 0.01). The interaction effect of the latent variables in sub-models 4–6 was significant, and further mediating effect analysis could be carried out.

##### Step 2: Analysis of Mediating Effect

According to the model shown in Figure 2, the independent variables, *X*, were changed to “*MM* ↔ *SM*”, “*MM* ↔ *FTF*” and “*SM* ↔ *FTF*”, respectively. The mediating effects of *IP* and *TP* were analyzed, and the results are shown in Table 7.

Sub-model 4: The relationship between MM*SM and *IP* is significant; the relationship between *IP* and *RAD* is not significant; the Slobe test shows that *Z_IP_* = 1.31 > 0.97, which is significant; the independent intermediary effect of *IP* is significant, and can inhibit the relationship between MM*SM and *RAD*; the relationship between *MM* *SM and *TP* is not significant; the relationship between *TP* and *RAD* is significant; the Slobe test shows that *Z_TP_* = 0.28 < 0.97, which is not significant; *TP* has no independent mediating effect; the overall mediating effect of *IP* and *TP* is not significant.

Sub-model 5: The relationship between MM*FTF and *IP* is not significant; the relationship between *IP* and *RAD* is significant; the Slobe test shows that *Z_IP_* = 0.19 < 0.97,which is not significant; the independent mediating effect of *IP* is not significant; the relationship between MM*FTF and *TP* is significant; the relationship between *TP* and *RAD* is not significant; the Slobe test shows that *Z_TP_* = 1.32 > 0.97, which is significant; the independent mediating effect of *TP* is significant, which can promote the relationship between MM*FTF and *RAD*; and the overall mediating effect of *IP* and *TP* was not significant.

Sub-model 6: The relationship between SM*FTF and *IP* is not significant; the relationship between *IP* and *RAD* is significant; the Slobe test shows that *Z_IP_* = 0.40 < 0.97, which is not significant; the relationship between SM*FTF and *TP* is not significant; the relationship between *TP* and *RAD* is not significant; the independent mediating effect of *TP* is not significant, and the overall effect of *IP* and *TP* is not significant.

In summary, the mediating effects of the mediator variables *IP*, *TP* are not significant from MM*SM, MM*FTF, and SM*FTF to make *RAD*, that is, hazard information transmitted by MM*SM, MM*FTF, and SM*FTF through the mediator variable *IP*, *TP*, cannot effectively promote the urban residents to make *RAD*, and communication channels of MM*SM, MM*FTF, and SM*FTF tend to establish a positive correlation with the *RAD* directly.

#### 3.2.3. Mediating Effect Analysis and Hypothesis Testing

After the analysis of the six models’ mediation effects, Hypothesis 1 was supported by the communication channels, and their combinations can be directly established with the *RAD*; Hypothesis 2 was supported by the relationship between *MM*, *SM* and *TP* which is significant, and as the relationship between *FTF* and *TP* is not significant, *MM* and *SM* can be established directly with *TP*, and not through the *IP*; Hypothesis 3 was not supported by the relationship between *IP* and *RAD*, which is shown in sub-models 1–4 as not significant; Hypothesis 4 was not supported by Table 7 which shows that *IP* and *TP* do not have a mediating effect in sub-models 4–6.

**Figure 3:**
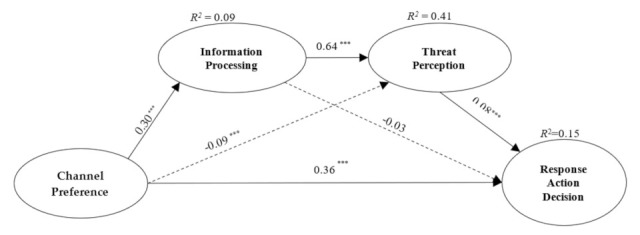
Schematic diagram of multi-stage correction model. Note: *** represents *p* < 0.001, the dash line indicates that there is no effective link between the two variables.

*X* in Figure 2 will be replaced by the *Channel Preference* whose measurement indexes are *MM*, *SM*, and *FTF*. In this paper, the measurement index values of three communication channels are centered and averaged as the measurement values of *MM*, *SM*, and *FTF*, with the overall analysis results of the model shown in Figure 3. Among them, *IP* cannot establish a valid relationship with the *RAD*; *Channel Preference* and *TP* have a negative correlation and, given the two values are too small, must be ignored.

### 3.3. Channel Preference Analysis

#### 3.3.1. Channel Preference Analysis of Sub-Model 1

In order to further understand the specific differences in urban residents’ preferences for different channels under the mediating effect of *IP* and *TP*, they are analyzed by gender, age, education, marital status, geographical location, income, and experience. In the structural equation model, the relationship between the independent variable and the dependent variable can be analyzed by using gender, age, and other factors as moderator variables. Since the moderator variables represented by the above factors are the categorical variables, and the independent variables are latent variables, the method of multi-group analysis is used [71]. As the goal of this study is to analyze whether the population of different demographic characteristics have different channel preferences for the theoretical models, it is necessary to parameterize the unconstrained model. In order to make the research more targeted, in constrained model1 the measurement weights are equal, in constrained model 2 the measurement intercepts are equal, in constrained model 3 the structural weights are equal. The constraints of the constrained model are enhanced in turn, by comparing the critical ratios for differences between parameters to determine the significant differences in the different groups for the model. After multi-group analysis of the different demographic characteristics, it was found that the fitting indicators of different constrained models are in line with the requirements. The results showed that: *CMIN*/*DF* was less than 3, while *NFI*, *RFI*, *IFI*, *TLI*, *CFI* were more than 0.9, and *RMSEA* was less than 0.5. The significant differences can be further compared to analyze the channel preferences and the moderating effect of the different demographic characteristics.

The results of the sub-model multi-group analysis show that:

- Sex group: comparing critical ratios for differences between parameters showed that there was no significant difference between the sex groups for the path *MM*→*IP*→*TP*→*RAD*; male ($a_{1}a_{2}a_{3}=0.015$) were more preferred than female ($a_{1}a_{2}a_{3}=0.006$).
- Marriage group: there were significant differences between the unmarried group ($a_{1}a_{2}a_{3}=0.025$) who are more inclined to *MM*→*IP*→*TP*→*RAD* than the married group ($a_{1}a_{2}a_{3}=0.007$); the divorced group tends to *MM*→*RAD* (*p*_a1_ > 0.05); the married (*β* = 0.272, *p* < 0.001), unmarried (*β* = 0.417, *p* < 0.001) and divorced (*β* = 0.842, *p* > 0.05) groups have significant differences for the path *MM*→*RAD*.
- Age group: there were significant differences between the 15–29-year-olds and 45–59 age groups who were more likely to *MM*→*IP*→*TP*→*RAD*; the 30–44-year-olds (*p*_a3_ > 0.05) group and the 60–74-year-olds group (*p*_a1_, *p*_a2_, *p*_a3_ > 0.05) were more inclined to *MM*→*RAD*.
- Education group: there were significant differences between the junior high school and below group ($a_{1}a_{2}a_{3}=0.003$), who were more inclined to *MM*→*IP*→*TP*→*RAD* than the university group ($a_{1}a_{2}a_{3}=0.005$); the high school group and master’s degree group tend to *MM*→*RAD*.
- Income group: there were significant differences between the L1.5 group ($a_{1}a_{2}a_{3}=0.023$), who were more inclined to *MM*→*IP*→*TP*→*RAD* than the 1.5–2.5 group ($a_{1}a_{2}a_{3}=0.017$); 2.5–4.5, 4.5–6.5, m6.5 three groups (*p*_a3_ > 0.05) prefer *MM*→*RAD*.
- Experience group: There was no significant difference in path *MM*→*IP*→*TP*→*RAD*, and the no disaster training experience group ($a_{1}a_{2}a_{3}=0.009$) was more inclined to *MM*→*IP*→*TP*→*RAD* than the group which has the disaster training experience ($a_{1}a_{2}a_{3}=0.008$).
- Geographical location group: There were significant differences in the regression coefficients between *MM* and *RAD* in the Chongqing area and Tianjin area; Changzhou area and Zibo area, and Tianjin residents prefer *MM*→*IP*→*TP*→*RAD*.

Summary 1: channel preference features of *MM*: male or female, unmarried or married, 15–29-year-olds, junior secondary education, L1.5 income, no disaster experience, Tianjin area; the gender and experience group for the theoretical model with independent variable *MM* had an excellent moderator effect, other demographic characteristics due to existing significant differences, cannot achieve cross-group impact, so there was no moderator effect.

#### 3.3.2. Channel Preference Analysis of Sub-Model 2

The results of the sub-model 2 multi-group analysis show that:

- Sex group: there were significant differences, where the male group ($a_{1}a_{2}a_{3}=0.014$) was more inclined to *SM*→*IP*→*TP*→*RAD*, while the female group tended to *SM*→*RAD* (*p*_a1_ > 0.05).
- Marriage group: there were significant differences, i.e., the unmarried group ($a_{1}a_{2}a_{3}=0.025$) was more inclined to *SM*→*IP*→*TP*→*RAD* than the married group ($a_{1}a_{2}a_{3}=0.004$); the divorced group tended to *SM*→*RAD* (*p*_a1_ > 0.05).
- Age group: there were significant differences: the 15–29-year-old ($a_{1}a_{2}a_{3}=0.017$) group were more likely to *SM*→*IP*→*TP*→*RAD*; the 30–44 year old (*p*_a1_, *p*_a3_ > 0.05), 45–59-year-old (*p*_a1_ > 0.05), 60–74-year-old (*p*_a1,_ *p*_a2,_ *p*_a3_ > 0.05) groups were more inclined to *SM*→*RAD*.
- Education group: there were significant differences: junior high school and below groups ($a_{1}a_{2}a_{3}=0.041$) were more inclined towards *SM*→*IP*→*TP*→*RAD*; the high school group (*p*_a1_, *p*_a3_ > 0.05), university group (*p*_a1_, *p*_a3_ > 0.05), and master’s degree group (*p*_a1_, *p*_a3_ > 0.05) tend to *SM*→*RAD*.
- Income group: there were significant differences: the 1.5–2.5 group ($a_{1}a_{2}a_{3}=0.016$) was more inclined towards *SM*→*IP*→*TP*→*RAD*; the L1.5, 2.5–4.5, 4.5–6.5, m6.5 four groups (*p*_a3_ > 0.05) preferred *SM*→*RAD*.
- Experience group: there were significant differences: the group having disaster training experience ($a_{1}a_{2}a_{3}=0.007$) was more inclined to *SM*→*IP*→*TP*→*RAD*; the group with no disaster training experience group tended toward *SM*→*RAD* (*p*_a1_ > 0.05).
- Geographical location group: significant difference existed, Lanzhou residents ($a_{1}a_{2}a_{3}=0.024$) were more inclined to *SM*→*IP*→*TP*→*RAD* than Zhangzhou ($a_{1}a_{2}a_{3}=0.016$); Chongqing (*p*_a1_ > 0.05), Tianjin (*p*_a1_, *p*_a3_ > 0.05), Changzhou (*p*_a3_ > 0.05), Zibo (*p*_a1_, *p*_a3_ > 0.05) residents tend to *SM*→*RAD*.

Summary 2: channel preference features of *SM*: male, unmarried or married, 15–29-year-old, junior secondary education, 1.5–2.5, having training experience, Lanzhou or Zhangzhou area; different demographic characteristics due to existing significant differences, cannot achieve the cross-group effect, so there is no moderator effect.

#### 3.3.3. Channel Preference Analysis of Sub-Model 3

Sub-model 3 multi-group analysis results show that:

- *Sex group:* the differences are significant: the male group ($a_{1}a_{2}a_{3}=0.025$) was more inclined to *FTF*→*IP*→*TP*→*RAD*, and the female group tends to *FTF*→*RAD* (*p*_a1_ > 0.05).
- *Marriage group:* the differences were significant: the unmarried group ($a_{1}a_{2}a_{3}=0.046$) was more inclined to *FTF*→*IP*→*TP*→*RAD* than the married group ($a_{1}a_{2}a_{3}=0.012$), the divorced group tend to *FTF*→*RAD* (*p*_a1_ > 0.05).
- *Age group:* the differences were significant: the 15–29-year-old group ($a_{1}a_{2}a_{3}=0.017$) liked *FTF*→*IP*→*TP*→*RAD better*; the 30–44 year old (*p*_a1_, *p*_a3_ > 0.05), 45–59-year-old (*p*_a1_ > 0.05), 60–74-year-old (*p*_a1,_ *p*_a2,_ *p*_a3_ > 0.05) groups were more inclined to *FTF*→*RAD*.
- *Education group:* the differences were significant: the junior high school and below group ($a_{1}a_{2}a_{3}=0.058$) was more inclined to *FTF*→*IP*→*TP*→*RAD*; the high school group (*p*_a3_ > 0.05), university group (*p*_a3_ > 0.05), and master’s degree group (*p*_a3_ > 0.05) preferred *FTF*→*RAD*.
- *Income group*: the differences were significant: the 1.5–2.5 group ($a_{1}a_{2}a_{3}=0.067$) was more inclined to *FTF*→*IP*→*TP*→*RAD*; the L1.5, 2.5–4.5, 4.5–6.5, m6.5 four groups (*p*_a3_ > 0.05) preferred *FTF*→*RAD*.
- *Experience group*: the differences were significant: the group having disaster training experience ($a_{1}a_{2}a_{3}=0.026$) was more inclined towards *FTF*→*IP*→*TP*→*RAD*; the group with no disaster training experience tends to *FTF*→*RAD* (*p*_a3_ > 0.05).
- *Geographical location group*: the differences were significant: Lanzhou residents ($a_{1}a_{2}a_{3}=0.046$) were more inclined to *FTF*→*IP*→*TP*→*RAD* than Zhangzhou ($a_{1}a_{2}a_{3}=0.023$) and than Chongqing ($a_{1}a_{2}a_{3}=0.017$; Tianjin (*p*_a3_ > 0.05) and Changzhou (*p*_a3_ > 0.05), Zibo (*p*_a3_ > 0.05) residents favored *FTF*→*RAD*.

Summary 3: channel preference features of *FTF*: male, unmarried or married, 15–29-year-old, junior secondary education, 1.5–2.5, having training experience, Lanzhou or Zhangzhou or Chongqing area; different demographic characteristics due to existing significant differences, cannot achieve the cross-group effect, so there is no moderator effect.

Given that the relationship between the independent variable and the *RAD* is significant and there is a negative correlation in sub-models 4–6, that is, the hazard information transmitted from the combination of different channels has a particular effect on the urban residents making *RAD*. Therefore, the study no longer analyzes the preferences of urban residents’ channel combinations with different demographic characteristics.

#### 3.3.4. Channel Preference Comprehensive Analysis of Sub-Models 1–3

After analyzing the channel preference of sub-models 1–3, it can be derived from the tendency of groups with different characteristics on the same channel. In order to further analyze the preferences of the same group for different channels, the results of the sub-models 1–3 should be combined to compare the different models. The results are shown in Table 8.

Table 8 shows that the overall effect of hazard information on hazardous chemicals transmitted by *MM* to promote urban residents to make *RAD* is better than *SM*, and better than *FTF*; the results between different channels show that men, married, unmarried, 15–29-year-olds, junior high school, and 1.5–2.5 groups are all inclined to the three channels; divorced, 30–44-year-olds, 60–74-year-olds, high school, master’s degree, 2.5–4.5,4.5–6.5, M6.5, and the Changzhou and Zibo regional groups do not prefer three channels; women, 45–59-year-olds, university, income less than 1.5, disaster training experience, Tianjin community groups prefer *MM*→*IP*→*TP*→*RAD*; the group having disaster training experience, Zhangzhou, and Lanzhou groups also prefer *SM*→*IP*→*TP*→*RAD*; the group with no disaster training experience, Chongqing, Zhangzhou, Lanzhou groups prefer *FTF*→*IP*→*TP*→*RAD*.

#### 3.3.5. Channel Preference Analysis and Hypothesis Testing

Hypothesis 5 was partially supported by results of sub-models 1–3 showing that men prefer *MM*, *SM*, *FTF*; females prefer *MM*; men tend to *FTF*→*IP*→*TP*→*RAD*; Hypothesis 6 was partially supported by results showing that married and unmarried prefer *MM*, *SM*, *FTF*→*IP*→*TP*→*RAD*, and the divorced group prefers *SM*; Hypothesis 7 was partially supported by results proving that the 15–29-year-olds prefer three channels; the 45–59-year-olds prefer *FTF*→*IP*→*TP*→*RAD*; the 30–44-year-olds and 60–74-year-olds prefer *SM*→*RAD*; Hypothesis 8 was partially supported by different educational groups who have different channel preferences, such as the junior high school group prefers *FTF*→*IP*→*TP*→*RAD* and the university group prefers *MM*→*IP*→*TP*→*RAD*; Hypothesis 9 was partially supported by different income groups having different channel preferences, such as the 1.5–2.5 prefers *FTF*→*IP*→*TP*→*RAD*; Hypothesis 10 was partially supported by results showing that the group having experience prefers *MM*→*IP*→*TP*→*RAD*, while the group with no experience group prefers *FTF*→*IP*→*TP*→*RAD*; Hypothesis 11 was partially supported by results showing Chongqing residents favor *FTF*→*IP*→*TP*→*RAD*, while Tianjin residents prefer *MM*→*IP*→*TP*→*RAD*; Hypothesis 12 was supported by the existence of different channels→*IP*→*TP*→*RAD*, and that different groups with different characteristics select different channels→*IP*→*TP*→*RAD*, proving that *RADM* could be better applied to analysis and research on threat perception of hazardous chemicals and response action decision of urban residents in China.

## 4. Discussion

### 4.1. Mediating Effect

In the multi-stage model of response action decisions for hazardous chemicals, *IP* and *TP* serve as two-stage mediator variables, which play an orderly role in a series of model operations. The results of sub-models 1–3 show that *IP* has a partial mediating effect in sub-models 1–2, and the mediating effect of *IP* in sub-model 2 is larger than that of sub-model 1; *IP* and *TP* have a complete mediation effect on sub-model 3; in the three sub-models, the two mediator variables show the characteristics of the partial mediating effect. The details are as follows:

The mediating effect of *IP* and *TP* on the extent of promoting urban residents to make *RAD* through *FTF* (27.5%) is better than that of *MM* (22.2%), and better than *SM* (12.2%). This result is related to the inherent requirements of *IP* and *TP*, as well as the transmission characteristics of the *FTF* channel. As the definition of the mediator variable, *IP* and *TP* require the urban residents to fully absorb, digest and understand the information obtained and turn it into a well-defined threat perception, so that they can have clearer cognition about the probability, consequence, and control method for the occurrence of the hazard. As *FTF* requires a strong interaction, urban residents can either ask managers or advocates when faced with problems they cannot understand. Therefore, it is the most important measure to influence the transmission effect of *FTF*. However, *FTF* has no memory repeatability compared to *MM* and *SM*, that is, for the degree of understanding of the information transmitted, the ability to immediately grasp the essence can be converted into *RAD*. For example, if you do not immediately understand, you need to consult training companions, managers or advocates again. For organizations in community must be reported to and approved by the authorities, the process can become lengthy, and therefore organizers want urban residents to participate in the training as much as possible to master the relevant knowledge and practice the relevant skills. This also forces urban residents adopting *FTF* channels to obtain information about hazardous chemicals, in order to have a deeper understanding of the hazard they pose. The difficulty of using the *FTF* channel to communicate hazard information is that it encourages a preference for *D* as a method of obtaining hazard information in urban residents, which must go through *IP* to enhance *TP*.

Among the measurement indexes of *IP*, the “exposure” index has the greatest influence, while the most influential measure of *TP* is “serious”. The possibility of receiving harmful information, and the perception of the serious consequences of the disaster, have become key factors in the transmission of hazard information that can effectively improve people’s ability to make *RAD*. This can be explained, on the one hand in terms of hazard information on hazardous chemicals, where there are currently few communication channels, which prevents urban residents from effectively obtaining relevant information. On the other hand, urban residents lack knowledge on the serious consequences brought by hazardous chemicals disasters. The reason for this is because there is relatively little exposure of the chemical production industry in China, meaning that residents would need a certain basis to understand the technical terms. Urban residents with fast-paced lives do not have a great patience to query, learn and master the knowledge around hazardous chemicals, knowledge reserves are scarce, and when chemical production accidents occur, the government departments tend to block the news in order to avoid unnecessary panic. Mainstream media reports are more focused on the causes of the accident, casualties caused by the accident, and process by which the accident is handled. When urban residents are more concerned about the number of casualties, follow-up effects such as the attitude of Government in handling the accident, personnel mobilization, family appeasement and other initiatives which can be used as gossip material, they are less concerned about how accidents occur or how to avoid the reoccurrence of similar incidents in their daily lives.

### 4.2. Channel Preference

Different channels have different communication characteristics. In relation to *MM*: information on the threats posed by hazardous chemicals spread by *M* is based on the mode of “you speak, I listen; you write, I read”, which lacks interactivity (Xiao, Xu, Wang, 2016) [83]. Limited layout and space prevent comprehensive reports, as a large number of terms or professional terminology which cannot be understood by general residents get mixed in with the information, forcing the passive absorption of information and preventing active learning. *SM* spreads information faster, with the Ministry of Industry and Information Technology data showing that the number of mobile internet users in China has exceeded 1.1 billion, with the total number of mobile phone users in China reaching 1.36 billion [84]. We-Chat, a daily chat app, plays a huge role in information dissemination. As its user base is very large, its convenience makes social media the primary method for urban residents to obtain hazard information. On the other hand, the lack of gatekeeping also leads to the disadvantage of inadequate authority for *SM*. The open network allows people to freely release information, including a large amount of false information, which in turn reduces residents’ trust in *SM* (Zhao, 2016) [85]. In terms of *FTF*, expert explanation, theoretical lectures, and other *FTF* forms have a more intuitive understanding, and their interactivity means that they are easily accepted by urban residents. The hazard information transmitted by *FTF* has integrated the orientation of managers and social mainstream public opinion, with the characteristic of incomplete content coverage. However, the form of transmission of *FTF* does not benefit from the active absorption of the audience, especially in training seminars, where many audiences attend under pressure, and if the contents of training seminars cannot be effectively mastered, communication effectiveness is impacted [86,87]. Different demographic groups choose different channels to obtain hazard information and make *RAD*. This paper discusses not only the different demographic groups having the same channel preferences, such as males, married and unmarried groups having a certain tendency for *MM*, *SM*, and *FTF*, but also discusses differences in channel preference and the reasons for these amongst different demographic groups.

While the no disaster experience group prefers *MM* and *FTF*, the group having disaster experience prefers *MM* and *SM*. Because the group with disaster experience has a certain amount of disaster experience and the ability to identify hazard information, the reliability of information sources and fast & convenient communication channels have become the first choice for this group. In contrast, the no disaster experience group needs to repeatedly check the authenticity of information, the consequences of the disaster occurrence and other relevant information, before making the corresponding *RAD*. Because females have relatively less working pressure, and have more time to take care of children and families, the way they spend free time tends to be watching TV at home, which is why the hazard information transmitted by *MM* can promote female groups to make *RAD*. As for why the 45–59-year-old group prefers *MM*, as the group mainly includes occupations such as positions inside government agencies, institutions or state-owned enterprises, professional characteristics and internal requirements (sitting office, confidentiality), the group has more contact with *MM* in daily life, and so become more inclined to trust the authority of *MM*. The university group is concentrated in the three age groups of 15–29-year-olds, 30–44-year-olds, and 45–59-year-olds, of which 70.6% have disaster training experience and, therefore, the authority of the information source has a greater impact. Among the groups with income less than 15,000, 74.2% are students, and 63.4% are college students, so the channel preference of this group is similar to that of the university group. Channel preferences differ in different regions, mainly due to local cultural traditions and the leadership style of the local government.

## 5. Conclusions

Combined with the theory and application of *RADM*, this paper puts forward a theoretical model of response action decision in relation to hazardous chemicals. The analysis on the mediating effect of the mediator variables *IP* and *TP* in the model shows that:

1. For the multi-stage model of channel preferences *MM*, *SM*, *FTF*, and both *IP* and *TP* have a significant mediating effect, which can promote *RAD* by channel preference→*IP*→*TP*.
2. For the channel combination “*MM*↔*SM*”, the mediating effect of *IP* and *TP* is significant, and can have a certain inhibitory effect; *IP* and *TP* have no mediating effect in the channel combinations “*MM*↔*FTF*” and “*SM*↔*FTF*”, in other words, *IP* and *TP* have no positive effect on the decision model of the channel combination as independent variables.
3. Hypothesis 1 is valid, Hypothesis 2 is valid, Hypothesis 3 is not valid, and Hypothesis 4 is not valid.

In order to further verify the mediator variables, the paper studies whether *IP* and *TP* have a mediating effect for different characteristics groups in obtaining hazard information and making *RAD* through different channels; moreover, this paper analyzes the channel preferences of different characteristics groups. Research results show:

4. Overall effect of the channel *MM* is better than *SM*, and better than *FTF*;
5. Male, married, unmarried, 15–29-year-old, junior high school, 1.5–2.5 groups are inclined to three channels, that is, the mediating effect of *IP* and *TP* is significant for these groups;
6. Divorced, 30–44-year-old, 60–74-year-old, high school, master’s degree, 2.5–4.5,4.5–6.5, M6.5, Changzhou, and Zibo regional groups are inclined to three channels, that is, the mediating effect of *IP* and *TP* is not significant for these groups;
7. While female, 45–59-year-old, university, income less than 1.5, disaster training experience, Tianjin resident groups prefer *MM*→*IP*→*TP*→*RAD*, the group having disaster training experience, Zhangzhou, and Lanzhou groups favor *SM*→*IP*→*TP*→*RAD*; the group having no disaster training experience, Chongqing, Zhangzhou, and Lanzhou groups prefer *FTF*→*IP*→*TP*→*RAD*, that is, *IP* and *TP* produce a mediating effect on these groups which need to go through specific channels;
8. While Hypotheses 4—11 are partially valid, Hypothesis 12 is valid. While integrating the results of the mediating effect and channel preference analysis, *RADM* can be used effectively to study how Chinese urban residents can improve threat perception and make response action decisions.

## Figures and Tables

**Table 1:** Description of variables.

Variable Type	Variable Name	Measurement Index	Index Explanation
Independent variable	*Mass media* (*MM*) *Social media* (*SM*) *Face to face* (*FTF*)	preference	Whether more likely to choose this channel to obtain hazard information
		permeability	The extent to which daily life is disturbed by hazard information
		accuracy	The authority of hazard information dissemination
		stability	The extent to which hazard information is understood
Mediator variable	*Information processing* (*IP*)	exposure	Possibility of obtaining hazard information
		concern	Degree of concern around hazard information
		comprehension	Degree of understanding of hazard information.
Mediator variable	*Threat perception* (*TP*)	possibility	The likelihood of accident to occur around hazardous chemicals
		seriousness	Threat to life, safety and property security.
		fear and unknown factors	Unknown and degree of fear of the characteristics of the hazards and evacuation routes etc.
Dependent variable	*Response Action* *Decision (RAD)*	risk identification	Is there a real threat that I need to pay attention to?
		risk assessment	Do I need to take hazard response action?
		hazard response action search	What can be done to achieve hazard response?
		hazard response action assessment	What is the best method of hazard response?
		hazard response action implementation	Does hazard response action need to be taken now?
		information-seeking activities	What information do I need to answer my question?

Note: MM represents mass media, SM represents social media, FTF represents face-to-face communication, IP represents information processing, TP represents threat perception, and RAD represents Hazard Response Action Decision (the same below).

**Table 2:** Resident population and sample size of six cities.

City	Resident Population	Sample Size
Chongqing	30,170,000	246
Tianjin	15,470,000	246
Zhangzhou	5,000,000	246
Changzhou	4,701,000	246
Zibo	4,642,000	246
Lanzhou	3,679,000	246

**Table 3:** Demographic Data of the Participants (*n* = 1700).

Variable		*n*	%
Gender	Male	826	48.6
	Female	874	51.4
Marriage status	Married	1135	66.8
	Unmarried	508	30.0
	Divorced	50	2.8
	Widowed	7	0.4
Age	Less than 15	3	0.2
	15–29 year old	536	31.5
	30–44	560	32.9
	45–59	582	34.3
	60—74	17	1.0
	More than 75	2	0.1
Education	Junior high school and below	219	12.9
	High school or secondary school	451	26.5
	University	948	55.8
	Master’s degree or above	82	4.8
Income	Less than 15,000	236	13.9
	15,000–25,000	234	13.8
	25,000–45,000	337	19.7
	45,000–65,000	383	22.5
	More than 65,000	510	30.0
Hazards Experience	Yes	1116	65.6
	No	584	34.4

Note: the data groups for people who are widowed, aged less than 15, and aged more than 75 are too small to affect the operation of the model and the accuracy of the results, and are therefore too small to be used for characteristics analysis.

**Table 4:** Confirmatory Factor Analysis Results for Multi-stage Model.

Variables	Items	Factor Loading	*S.E*.	C.R.	*p*	*R* ^2^	Composite Reliability	*AVE*
*MM*	*MM1*	0.646	0.033	26.661	***	0.417	0.843	0.575
	*MM2*	0.722	0.031	30.352	***	0.521		
	*MM3*	0.832	0.030	35.275	***	0.693		
	*MM4*	0.818	*/*	*/*	*a*	0.670		
*SM*	*SM1*	0.742	0.034	26.079	***	0.550	0.817	0.531
	*SM2*	0.859	0.045	28.289	***	0.737		
	*SM3*	0.594	0.043	26.551	***	0.353		
	*SM4*	0.696	*/*	*/*	*a*	0.484		
*FTF*	*FTF1*	0.640	0.032	33.808	***	0.410	0.840	0.569
	*FTF2*	0.714	0.035	29.441	***	0.510		
	*FTF3*	0.842	0.037	26.017	***	0.709		
	*FTF4*	0.805	*/*	*/*	*a*	0.648		
*IP*	*IP1*	0.740	0.040	19.850	***	0.548	0.809	0.586
	*IP2*	0.759	0.042	19.129	***	0.576		
	*IP3*	0.796	*/*	*/*	*a*	0.664		
*TP*	*TP1*	0.738	0.053	11.273	***	0.545	0.838	0.639
	*TP2*	0.650	0.061	14.898	***	0.423		
	*TP3*	0.975	*/*	*/*	*a*	0.951		
*RAD*	*RAD1*	0.710	*/*	*/*	*a*	0.505	0.886	0.565
	*RAD2*	0.762	0.031	36.181	***	0.580		
	*RAD3*	0.811	0.042	29.903	***	0.657		
	*RAD4*	0.800	0.039	29.583	***	0.640		
	*RAD5*	0.705	0.037	26.308	***	0.497		
	*RAD6*	0.714	0.037	26.642	***	0.510		

Note: A representative regression weight was fixed at 1.0. The S.E. (standard error), C.R. (composite reliability), and *p*-value were not estimated in these cases. However, by fixing a different parameter, we determined that the estimates of these scaled values are also statistically significant with *p* < 0.01, *** stands for *p* < 0.001.

**Table 5:** The statistical results of sub-models 1–3.

Sub-Models		*Estimate*	*S.E*.	*C.R*.	*p*
*Sub-model 1*	*MM*→*IP*	0.206	0.030	6.794	***
	*IP*→*TP*	0.505	0.026	19.186	***
	*MM*→*TP*	**−0.057**	0.018	−3.200	0.001
	*TP*→*RAD*	0.112	0.044	2.574	0.010
	*MM*→*RAD*	0.242	0.022	10.811	***
	*IP*→*RAD*	0.010	0.037	0.278	**0.781**
*Sub-model 2*	*SM*→*IP*	0.144	0.035	4.081	***
	*IP*→*TP*	0.492	0.025	19.453	***
	*SM*→*TP*	**−0.051**	0.020	−2.559	0.011
	*TP*→*RAD*	0.186	0.048	3.872	***
	*SM*→*RAD*	0.215	0.025	8.498	***
	*IP*→*RAD*	**−0.056**	0.040	−1.374	**0.169**
*Sub-model 3*	*FTF*→*IP*	0.385	0.037	10.308	***
	*IP*→*TP*	0.501	0.027	18.403	***
	*FTF*→*TP*	**−0.035**	0.022	−1.572	**0.116**
	*TP*→*RAD*	0.171	0.048	3.566	***
	*FTF*→*RAD*	0.161	0.028	5.754	***
	*IP*→*RAD*	**−0.073**	0.043	−1.671	**0.095**

Note: The bold font in the Estimate column indicates a negative correlation between the two variables; the bold font in the *p* column indicates that the relationship between the two variables is not significant, *** stands for *p* < 0.001.

**Table 6:** Mediation effect analysis of sub-models 1–3.

Model	*X*	$a_{1}b_{1}$	$a_{2}b_{2}$	$a_{1}a_{3}b_{2}$	$c^{\prime }$	$\left|a_{1}b_{1}+a_{2}b_{2}+a_{1}a_{3}b_{2}\right|$	$\left|a_{1}b_{1}+a_{1}a_{3}b_{2}\right|/$ $\left|a_{1}b_{1}+a_{2}b_{2}+a_{1}a_{3}b_{2}\right|$	$\left|a_{2}b_{2}+a_{1}a_{3}b_{2}\right|/$ $\left|a_{1}b_{1}+a_{2}b_{2}+a_{1}a_{3}b_{2}\right|$	$\left|a_{1}b_{1}+a_{2}b_{2}+a_{1}a_{3}b_{2}\right|/c$
Sub-model 1	*MM*	0	0.057	0.012	0.242	0.069	17.4%	100%	22.2%
Sub-model 2	*SM*	0.008	0.009	0.013	0.215	0.030	70.0%	73.3%	12.2%
Sub-model 3	*FTF*	0.028	0	0.033	0.161	0.061	100%	54.1%	27.5%

Note: *c* represents the total effect of the model, $\left|a_{1}a_{2}a_{3}\right|/c$ represents the ratio of the mediating effect to the total effect of the model.

**Table 7:** Mediation effect analysis of sub-models 4–6.

Sub-Models		Estimate	S.E.	C.R.	*p*
*Sub-model 4*	MM*SM→*IP*	−0.055	0.026	−2.103	0.035
	*IP→TP*	0.434	0.032	13.651	***
	MM*SM→*TP*	0.004	0.014	0.315	0.753
	*TP→RAD*	−0.103	0.018	−5.825	***
	MM*SM→RAD	0.090	0.061	1.479	0.139
	*IP→RAD*	0.065	0.039	1.660	0.097
*Sub-model 5*	MM*FTF→IP	−0.014	0.023	−0.628	0.530
	*IP→TP*	0.495	0.026	19.384	***
	MM*FTF→TP	0.026	0.013	2.051	0.040
	*TP→RAD*	0.076	0.043	1.776	0.076
	MM*FTF→RAD	−0.073	0.016	−4.570	***
	*IP→RAD*	0.083	0.037	2.251	0.024
*Sub-model 6*	SM*FTF→IP	0.013	0.032	0.418	0.676
	*IP→TP*	0.495	0.026	19.348	***
	SM*FTF→TP	0.024	0.018	1.329	0.184
	*TP→RAD*	0.068	0.042	1.609	0.108
	SM*FTF→RAD	−0.069	0.022	−3.092	0.002
	*IP→RAD*	0.090	0.037	2.450	0.014

Note: *** represents *p* < 0.001.

**Table 8:** Channel preference comprehensive analysis of sub-models 1–3.

Demographic Characteristics	Sub-Model 1		Sub-Model 2		Sub-Model 3	
	Tendency of *MM→IP→TP→RAD*	*MM*→*RAD*	Tendency of *SM*→*IP*→*TP*→*RAD*	*SM*→*RAD*	Tendency of *FTF*→*IP*→*TP*→*RAD*	*FTF*→*RAD*
Male	Obvious $a_{1}a_{2}a_{3}=0.015$	*β* = 0.335 ***	Obvious $a_{1}a_{2}a_{3}=0.014$	*β* = 0.263 ***	Obvious $a_{1}a_{2}a_{3}=0.025$	*β* = 0.156 ***
Female	Obvious $a_{1}a_{2}a_{3}=0.006$	*β* = 0.329 ***	Not Obvious *p*_a1_ > 0.05	*β* = 0.252 ***	Not Obvious *p*_a3_ > 0.05	*β* = 0.214 ***
Married	Obvious $a_{1}a_{2}a_{3}=0.007$	*β* = 0.271 ***	Obvious $a_{1}a_{2}a_{3}=0.004$	*β* = 0.197 ***	Obvious $a_{1}a_{2}a_{3}=0.012$	*β* = 0.132 ***
Unmarried	Obvious $a_{1}a_{2}a_{3}=0.025$	*β* = 0.434 ***	Obvious $a_{1}a_{2}a_{3}=0.025$	*β* = 0.356 ***	Obvious $a_{1}a_{2}a_{3}=0.046$	*β* = 0.307 ***
Divorced	Not Obvious *p*_a1_ > 0.05	*β* = 0.295 ***	Not Obvious *p*_a1_ > 0.05	*β* = 0.139	Not Obvious *p*_a1_ > 0.05	*β* = 0.012
15–29-year-old	Obvious $a_{1}a_{2}a_{3}=0.054$	*β* = 0.367 ***	Obvious $a_{1}a_{2}a_{3}=0.017$	*β* = 0.283 ***	Obvious $a_{1}a_{2}a_{3}=0.017$	*β* = 0.215 ***
30–44-year-old	Not Obvious *p*_a3_ > 0.05	*β* = 0.304 ***	Not Obvious *p*_a1_, *p*_a3_ > 0.05	*β* = 0.221 ***	Not Obvious *p*_a1_, *p*_a3_ > 0.05	*β* = 0.205 ***
45–59-year-old	Obvious $a_{1}a_{2}a_{3}=0.020$	*β* = 0.295 ***	Not Obvious *p*_a1_ > 0.05	*β* = 0.230 ***	Not Obvious *p*_a1_ > 0.05	*β* = 0.132 **
60–74-year-old	Not Obvious *p*_a1_, *p*_a2_, *p*_a3_ > 0.05	*β* = 0.208 ***	Not Obvious *p*_a1_, *p*_a2_, *p*_a3_ > 0.05	*β* = 0.121	Not Obvious *p*_a1_, *p*_a2_, *p*_a3_ > 0.05	*β* = 0.011
Junior high school and below	Obvious $a_{1}a_{2}a_{3}=0.03$	*β* = 0.431 ***	Obvious $a_{1}a_{2}a_{3}=0.041$	*β* = 0.345 ***	Obvious $a_{1}a_{2}a_{3}=0.058$	*β* = 0.263 ***
High school or secondary school	Not Obvious *p*_a3_ > 0.05	*β* = 0.312 ***	Not Obvious *p*_a1_, *p*_a3_ > 0.05	*β* = 0.136 *	Not Obvious *p*_a3_ > 0.05	*β* = 0.135 *
University	Obvious $a_{1}a_{2}a_{3}=0.005$	*β* = 0.271 ***	Not Obvious *p*_a1_, *p*_a3_ > 0.05	*β* = 0.241 ***	Not Obvious *p*_a3_ > 0.05	*β* = 0.171 ***
Master’s degree or above	Not Obvious *p*_a1_, *p*_a3_ > 0.05	*β* = 0.473 ***	Not Obvious *p*_a1_, *p*_a3_ > 0.05	*β* = 0.464 ***	Not Obvious *p*_a3_ > 0.05	*β* = 0.263
L15000	Obvious $a_{1}a_{2}a_{3}=0.023$	*β* = 0.375 ***	Not Obvious *p*_a3_ > 0.05	*β* = 0.306 ***	Not Obvious *p*_a3_ > 0.05	*β* = 0.213 **
15,000–25,000	Obvious $a_{1}a_{2}a_{3}=0.017$	*β* = 0.369 ***	Obvious $a_{1}a_{2}a_{3}=0.016$	*β* = 0.259 ***	Obvious $a_{1}a_{2}a_{3}=0.067$	*β* = 0.257 ***
25,000–45,000	Not Obvious *p*_a3_ > 0.05	*β* = 0.370 ***	Not Obvious *p*_a3_ > 0.05	*β* = 0.279 ***	Not Obvious *p*_a3_ > 0.05	*β* = 0.148 *
45,000–65,000	Not Obvious *p*_a3_ > 0.05	*β* = 0.327 ***	Not Obvious *p*_a3_ > 0.05	*β* = 0.258 ***	Not Obvious *p*_a3_ > 0.05	*β* = 0.138 *
M65000	Not Obvious *p*_a3_ > 0.05	*β* = 0.248 ***	Not Obvious *p*_a3_ > 0.05	*β* = 0.171 ***	Not Obvious *p*_a3_ > 0.05	*β* = 0.175 ***
Yes	Obvious $a_{1}a_{2}a_{3}=0.008$	*β* = 0.305 ***	Obvious $a_{1}a_{2}a_{3}=0.007$	*β* = 0.241 ***	Not Obvious *p*_a3_ > 0.05	*β* = 0.192 ***
No	Obvious $a_{1}a_{2}a_{3}=0.009$	*β* = 0.361 ***	Not Obvious *p*_a1_ > 0.05	*β* = 0.266 ***	Obvious $a_{1}a_{2}a_{3}=0.026$	*β* = 0.155 **
Chongqing	Not Obvious *p*_a1_, *p*_a3_ > 0.05	*β* = 0.330 ***	Not Obvious *p*_a1_ > 0.05	*β* = 0.217 **	Obvious $a_{1}a_{2}a_{3}=0.017$	*β* = 0.075
Tianjin	Obvious $a_{1}a_{2}a_{3}=0.027$	*β* = 0.176 **	Not Obvious *p*_a1_, *p*_a3_ > 0.05	*β* = 0.182 **	Not Obvious *p*_a3_ > 0.05	*β* = 0.077
Zhangzhou	Not Obvious *p*_a3_ > 0.05	*β* = 0.318 ***	Obvious $a_{1}a_{2}a_{3}=0.016$	*β* = 0.259 ***	Obvious $a_{1}a_{2}a_{3}=0.023$	*β* = 0.180 **
Changzhou	Not Obvious *p*_a3_ > 0.05	*β* = 0.341 ***	Not Obvious *p*_a3_ > 0.05	*β* = 0.265 ***	Not Obvious *p*_a3_ > 0.05	*β* = 0.311 ***
Zibo	Not Obvious *p*_a1_, *p*_a3_ > 0.05	*β* = 0.398 ***	Not Obvious *p*_a1_, *p*_a3_ > 0.05	*β* = 0.230 ***	Not Obvious *p*_a3_ > 0.05	*β* = 0.196 **
Lanzhou	Not Obvious *p*_a3_ > 0.05	*β* = 0.438 ***	Obvious $a_{1}a_{2}a_{3}=0.024$	*β* = 0.351 ***	Obvious $a_{1}a_{2}a_{3}=0.046$	*β* = 0.267 ***

Note: *** represents *p* < 0.001, ** represents *p* < 0.01, * represents *p* < 0.05.

## Data Availability

All authors announce that the data presented in this paper is original and not inappropriately selected, manipulated, enhanced, or fabricated.
